# Impact of Hypocaloric Dietary Intervention on Phenotypic Presentations of Polycystic Ovary Syndrome (PCOS)

**DOI:** 10.3390/nu17132223

**Published:** 2025-07-04

**Authors:** Faith E. Carter, Brittany Y. Jarrett, Alexis L. Oldfield, Heidi Vanden Brink, Joy Y. Kim, Marla E. Lujan

**Affiliations:** 1 Division of Nutritional Sciences, Cornell University, Ithaca, NY 14850, USA; fec25@cornell.edu (F.E.C.); byj4@cornell.edu (B.Y.J.); alo49@cornell.edu (A.L.O.); jyk47@cornell.edu (J.Y.K.); 2 Department of Nutrition, Texas A&M University, College Station, TX 77840, USA; heidi.vandenbrink@ag.tamu.edu

**Keywords:** polycystic ovary syndrome, weight loss, lifestyle, diet

## Abstract

**Background/Objective:** Lifestyle intervention is recommended as first-line treatment for polycystic ovary syndrome (PCOS). This pilot study aimed to determine if a short-term hypocaloric dietary intervention induced changes in the phenotypic presentation of PCOS. **Methods:** Twenty women with PCOS and overweight/obesity participated in a 3-month hypocaloric dietary intervention with a 6-month follow-up. At pre-intervention, post-intervention, and follow-up, assessments of menstrual cycle status, hyperandrogenism, and polycystic ovarian morphology were performed, and PCOS phenotype status was determined using the following scale of decreasing severity: Phenotype A (ovulatory dysfunction, hyperandrogenism, and polycystic ovaries), Phenotype B (ovulatory dysfunction and hyperandrogenism), Phenotype C (hyperandrogenism and polycystic ovaries), or Phenotype D (ovulatory dysfunction and polycystic ovaries). **Results:** The participants lost 8 ± 3% of their initial body weight with the intervention (*p* < 0.001). Eight (40%) participants experienced a favorable shift in PCOS phenotype, while the remaining 12 (60%) participants had an unfavorable shift or no change. Changes in PCOS phenotype were primarily driven by reductions in menstrual cycle length (*p* = 0.010) and follicle number per ovary (*p* = 0.017), albeit no baseline clinical variable predicted a favorable-change PCOS presentation. At the 6-month follow-up (N = 12), weight was increased (*p* < 0.05), and seven participants (58%) had reverted to a more severe phenotype. **Conclusions:** Weight loss may provide temporary improvement in the phenotypic presentation of PCOS, yet sustained lifestyle change may be required to maintain these benefits.

## 1. Introduction

Polycystic ovary syndrome (PCOS) is a prevalent endocrine disorder affecting one in eight women of reproductive age globally [1]. PCOS has long been considered the leading cause of anovulatory infertility, yet there is now acknowledgement that PCOS imparts serious risk for other reproductive, metabolic, and psychological health complications [1,2]. In the case of metabolic health, PCOS is a risk factor for obesity and commonly presents alongside insulin resistance, dyslipidemia, and hypertension [3,4,5,6]. Accordingly, women with PCOS are at higher risk for metabolic syndrome, type 2 diabetes, cardiovascular disease, and possibly cardiovascular mortality [2,7,8]. Healthy lifestyle behaviors aimed at weight management are recommended for all women with PCOS across the lifespan to alleviate these heighten health risks [2]. In those with excess adiposity, lifestyle intervention aimed at weight loss can improve metabolic health and potentially offset any added contributions of obesity to reproductive, metabolic, and psychological dysfunction in PCOS [9,10].

Current practice guidelines support a broad clinical spectrum for PCOS [2]. Namely, PCOS is defined by the presence of two or more cardinal features, (1) ovulatory dysfunction, (2) hyperandrogenism, and (3) polycystic ovarian morphology, after excluding for other reasons for these symptoms [2]. Based on possible combinations of the cardinal features, PCOS can manifest as four distinct clinical phenotypes: (1) Phenotype A (ovulatory dysfunction + hyperandrogenism + polycystic ovaries); (2) Phenotype B (ovulatory dysfunction + hyperandrogenism + normal ovaries); (3) Phenotype C (hyperandrogenism + polycystic ovaries + regular menstrual cycles); and (4) Phenotype D (ovulatory dysfunction + polycystic ovaries + normal androgens) [8]. Phenotype A is the most common phenotype presenting to primary and specialized care centers [11]. Likewise, Phenotype A is the most widely identified in population-based studies [3]. Classic forms of PCOS (Phenotypes A and B) are associated with the greatest degree of reproductive and metabolic dysfunction, likely owing to the direct and detrimental impact of hyperandrogenism on multiple organ systems [12,13,14,15,16,17]. By contrast, the non-androgenic Phenotype D is associated with fewer metabolic health risks despite evidence of neuroendocrine dysfunction and ovarian dysmorphology [18,19,20,21,22]. Nonetheless, all women with PCOS demonstrate greater risk of metabolic dysfunction compared to age- and weight-matched normoandrogenic women with regular menstrual cycles [23,24]. This increased susceptibility to multiple health complications warrants life-long surveillance of PCOS cardinal features and their associated co-morbidities [2,25,26].

Consideration of PCOS symptomology in clinical practice can enable the judicious counseling of patients on their relative health risks. Likewise, tracking of phenotypic presentation can provide a tool for gauging the progression and/or severity of the condition across the life course and in response to treatment options. Pooled evidence supports favorable impacts of lifestyle intervention on the metabolic health of women with PCOS [2,27]. However, the degree to which these improvements translate to demonstrable changes in PCOS phenotype status is relatively unexplored [28,29]. To that end, the primary objective of this pilot study was to determine if weight loss induced by a short-term hypocaloric dietary intervention led to changes in the clinical presentation of PCOS and whether these changes in phenotypic severity are sustained 6 months post-intervention. The potential for any clinical, endocrine, or morphological factor to predict favorable changes in PCOS phenotype status with hypocaloric dietary intervention was also explored as a secondary aim.

## 2. Materials and Methods

### 2.1. Participants and Study Design

This study represents a retrospective analysis of pre- and post-data garnered from a single-arm, 3-month hypocaloric dietary intervention involving women with PCOS. The trial included an ancillary protocol wherein women consented to return 6 months after the end of the dietary intervention for a follow-up assessment. Study participants were recruited from the general population of Ithaca, NY, and surrounding areas. Participants were between the ages of 18–38 years with overweight or obesity (BMI ≥ 25.0 kg/m^2^) and a self-reported history of irregular menstrual cycles and/or a previous diagnosis of PCOS. Participants were deemed eligible for the clinical trial if they (1) were at the ready to lose weight stage validated by questionnaire [30], (2) had not taken hormonal contraceptives or fertility treatments in the previous two months, (3) were not intending pregnancy during the study, (4) had not undergone ovarian or bariatric surgery, and (5) were not currently taking medications known to impact appetite, reproductive hormone levels, or metabolic profile (i.e., statins, insulin sensitizers, weight loss medications, valproate). Additional exclusion criteria included abnormalities in prolactin, thyroid hormone, and 17-hydroxprogesterone production.

### 2.2. Hypocaloric Dietary Intervention and Data Collection

After a 1-month baseline assessment period, in which participants were encouraged to continue their usual lifestyle behaviors, participants began a 3-month hypocaloric dietary intervention. The intervention was delivered as a hypocaloric commercial meal plan (Nutrisystem^®^ D, Nutrisystem, Inc., Fort Washington, PA, USA) known to promote weight loss of 1–2 lbs per week. The plan consisted of four low glycemic index, portion-controlled, and reduced-calorie meal entrees per day, supplemented with fresh foods by the participant per individual preferences. The diet adhered to the USDA Dietary Guidelines, consisting of 50% carbohydrate, 25% protein, and 25% fat, and included 25–35 g of fiber and <2300 milligrams of sodium [31]. Throughout the dietary intervention, participants met with a nutritionist weekly to customize meal plans that estimated 1250–1500 calories per day and to address any challenges as they arose. Participants visited with researchers every other day for the duration of the 4-month study. At the end of the intervention, participants discussed their continued goals for dietary intake and weight management with the researchers. Six months after the end of the intervention, participants returned for a follow-up assessment.

At baseline (pre-intervention), end of intervention (post-intervention), and 6-month follow-up, participants visited the research unit for the following assessments: (1) review of menstrual cycle history in the past year, (2) physical exam to assess vitals, anthropometry, and degree of hirsutism (modified Ferriman–Gallwey scoring), (3) dual X-ray absorptiometry (DEXA) scan to evaluate body composition, (4) transvaginal ultrasonography to assess ovarian morphology, (5) fasting blood tests for reproductive hormones and metabolic status indicators, and (6) a 2 h, 75 g oral glucose tolerance test to evaluate glucoregulatory control. Timing of the assessments was standardized to the early follicular phase and/or a time when there was no sonographic or hormonal evidence of a recent or pending ovulation. Ultrasound scans were performed using a GE Voluson E8 Expert System and 6–12 MHz endovaginal transducer (GE Healthcare, Milwaukee, WI, USA), and measurements of ovarian volume and follicle number per ovary made post hoc by expert raters with strong inter-rater agreement (Intraclass Correlation Coefficient > 0.80).

### 2.3. PCOS Phenotype Definitions

At baseline, post-intervention and 6-month follow-up, participants were assigned to one of four phenotypes using criteria for PCOS status supported by the 2018/2023 International Evidence-based Guideline for the Assessment and Management of PCOS [1,2]: (1) Phenotype A (ovulatory dysfunction + hyperandrogenism + polycystic ovaries); (2) Phenotype B (Non-PCO: ovulatory dysfunction + hyperandrogenism + normal ovaries); (3) Phenotype C (regular menstrual cycles + hyperandrogenism + polycystic ovaries); and (4) Phenotype D (ovulatory dysfunction + normal androgens + polycystic ovaries). Ovulatory dysfunction was based on the presence of menstrual irregularity defined by menstrual cycles lengths ≥ 36 days or <9 menstrual cycles in the previous year. Hyperandrogenism was defined by either biochemical or clinical evidence. Total testosterone > 59.4 ng/dL and/or free androgen index > 5.5 was considered evidence of androgen excess. Thresholds for biochemical hyperandrogenism were based on an internal reference standard reflecting the 95th percentile of levels generated in a group of healthy reproductive age women with regular cycles. Clinical hyperandrogenism was defined by a modified Ferriman–Gallwey score ≥ 6 per practice guideline recommendations [2]. Polycystic ovaries were defined by a follicle number per ovary ≥ 25 and/or an ovarian volume ≥ 10 mL in at least one ovary based on an internal standard [32,33] and guideline recommendations, respectively.

### 2.4. Biochemical Analysis

Serum FSH and LH were measured in-house by chemiluminescence immunoassays (Immulite 2000; Siemens Medical Solutions Diagnostics, Deerfield, IL, USA). AMH was measured with an enzyme-linked immunosorbent assay at a commercial laboratory (Ansh Labs, Webster, TX, USA). Total testosterone was measured by liquid chromatography tandem mass spectrometry at a clinical chemistry laboratory affiliated with in the Centers for Disease Control and Prevention Hormone Standardization (HoST) Program (Brigham Research Assay Core Laboratory, Boston, MA, USA) [34]. SHBG was measured in-house by chemiluminescence immunoassays (Immulite 2000; Siemens Medical Solutions Diagnostics, Deerfield, IL, USA), and values were used alongside testosterone levels to calculate the Free Androgen Index [(total testosterone ÷ SHBG) × 100] [35]. Venous blood glucose and insulin were measured by standard glucometer (Accu-Check Aviva, Roche Diabetes Care, Inc., Indianapolis, IN, USA) and chemiluminescence immunoassay, respectively. The homeostasis model of insulin resistance (HOMA-IR) was calculated as (fasting glucose × fasting insulin) ÷ 22.5 [36]. All intra- and inter-assay variations were ≤10%.

### 2.5. Statistical Analysis

All statistical analyses were performed using JMP software V.17 (SAS Institute, Cary, NC, USA). Distributions for all clinical variables of interest were evaluated for normality with Shapiro–Wilk tests. Variables that were not normally distributed were log transformed where appropriate. Linear mixed-models (Fixed Effects: Time and Random Effects: Participant ID) with Tukey HSD tests were used to compare endpoints across pre-intervention, post-intervention, and follow-up. Our primary outcome was a shift in PCOS phenotype. Participants were classified as having favorable or unfavorable shifts in PCOS phenotype post-intervention. Favorable responders were those that transitioned to a less severe PCOS phenotype. By contrast, unfavorable responders were those that transitioned to a more severe phenotype or had no change in phenotype post-intervention. Comparisons of clinical features between favorable and unfavorable responders were performed with Student’s *t*-tests and Wilcox signed rank tests. Logistic regression was used to evaluate associations among favorable shifts in PCOS status with (1) pre-intervention clinical characteristics and (2) percent change in clinical characteristics post-intervention. Statistical significance was set at *p* < 0.05.

### 2.6. Ethics Statement

The Institutional Review Board (IRB) at Cornell University approved the clinical trial associated with the data used in this current analysis. The clinical trial was registered with ClinicalTrials.gov (NCT01785719). Written informed consent was obtained from all study participants in the trial.

## 3. Results

### 3.1. Participant Characteristics at Baseline, Post-Intervention, and Follow-Up

**Figure 1:**
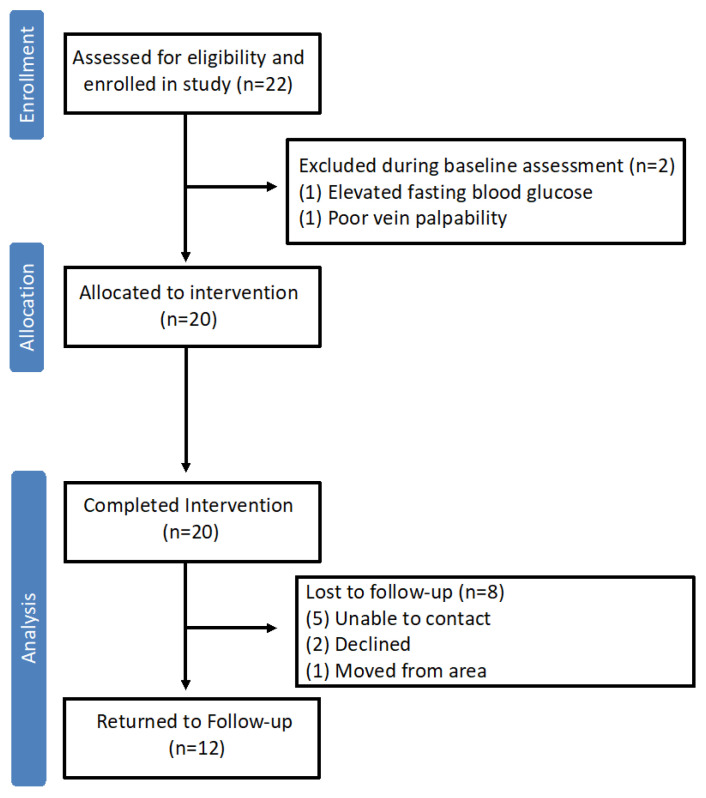
Flow of study participants through the original clinical trial involving a hypocaloric dietary intervention in women with PCOS. Of the 20 participants that proceeded to the hypocaloric dietary intervention, all completed the intervention and had sufficient clinical data at pre- and post-intervention timepoints for inclusion in the current analysis.

A schematic depicting the participant flow through the original clinical trial is presented in Figure 1. Twenty-two women with PCOS enrolled in the clinical trial, and all completed a baseline clinical assessment pre-intervention. Twenty participants were then allocated to the dietary intervention. All 20 participants completed the intervention and had a second clinical assessment of the end of the intervention. For one participant, the post-intervention assessment occurred 1 month earlier owing to unexpected travel. Ultimately, data from all 20 participants met inclusion criteria for the current pre-post analysis. Of the 20 participants that completed the post-intervention assessment, 12 returned 6 months later for a third clinical assessment (follow-up), including the individual that completed the post-intervention early. Sensitivity analysis excluding this participant’s data did not impact overall findings. Therefore, data from all the participants is included in the final analysis.

A comparison of anthropometric, body composition, and metabolic status markers at pre-intervention, post-intervention, and 6-month follow-up is provided in Table 1. Pre-intervention, the participants were on average 27 ± 5 years old and had a BMI of 37.5 ± 6.0 kg/m^2^, consistent with Class 2 (moderate-risk) obesity. Mean waist circumference (>88 cm), waist-to-hips ratio (>0.85), and total percent body fat (>30%) were elevated and outside healthy reference ranges for reproductive-age females [37,38]. Post-intervention, the participants had significantly lower weight, BMI, waist circumference, hips circumference, and waist-to-hips ratio compared to pre-intervention values (all *p* < 0.02). Significant reductions were also noted for body composition markers including total percent fat, total lean mass, and total trunk fat mass (*p* < 0.05), with the lean-to-fat mass ratio increased (*p* < 0.0001) relative to pre-intervention measures. Glucoregulatory status markers including fasting glucose, fasting insulin, HOMA-IR, and glucose and insulin levels at 2 h post-glucose ingestion were unchanged post-intervention (all *p* > 0.05). Likewise, systolic (*p* = 0.158) and diastolic pressure (*p* = 0.064) were similar pre- versus post-intervention. At the 6-month follow-up evaluation, weight, BMI, waist and hips circumference, and total lean mass were significantly higher compared to the post-intervention measures (all *p* < 0.01). However, weight, BMI, waist circumference, waist-to-hips ratio, and lean-to-fat mass ratio at the follow-up were still significantly lower compared to measures at the pre-intervention stage (all *p* < 0.025). Blood pressure and glucoregulatory status markers at the follow-up were similar to those at both pre- and post-intervention (*p* > 0.05).

The cardinal features of PCOS, alongside other reproductive status markers, are contrasted at pre-intervention, post-intervention, and 6-month follow-up in Table 2. Before the intervention, the average self-reported menstrual cycle length for the group met the criteria for amenorrhea (>90 days between menstrual periods). Menstrual cycle length significantly decreased post-intervention (*p* = 0.010), with 30% of the participants now demonstrating regular menstrual cyclicity (<36 days between menstrual periods). Average hirsutism scores reflected the presence of mild hirsutism at pre-intervention, and scores were unchanged in the post-intervention period (*p* = 0.752). Levels of total testosterone (*p* = 0.001) and sex hormone binding globulin (SHBG) were higher post-intervention (*p* = 0.012) and offset any potential increase in free androgen index (FAI), whose values were similar pre- versus post-intervention (*p* = 0.065). Average measures of FNPO and OV at pre-intervention met criteria for follicle excess (≥25 follicles) and ovarian enlargement (≥10 mL), respectively. Mean FNPO was decreased post-intervention (*p* = 0.017), albeit counts were still consistent with follicle excess. Likewise, average measures of OV were unchanged post-intervention (*p* = 0.624), with values reflecting persistence of ovarian enlargement. At the 6-month follow-up, menstrual cycle length (*p* = 0.645), hirsutism scores (*p* = 0.339), total testosterone (*p* = 0.931), and free androgen index (*p* = 0.879) were similar to the post-intervention measures, with total testosterone levels remaining significantly higher than those at the pre-intervention stage (*p* = 0.016). Likewise, measures of ovarian morphology at the follow-up were similar to those at pre- and post-intervention (all *p* > 0.05).

### 3.2. PCOS Phenotypes at Pre-Intervention, Post-Intervention, and Follow-Up

**Figure 2:**
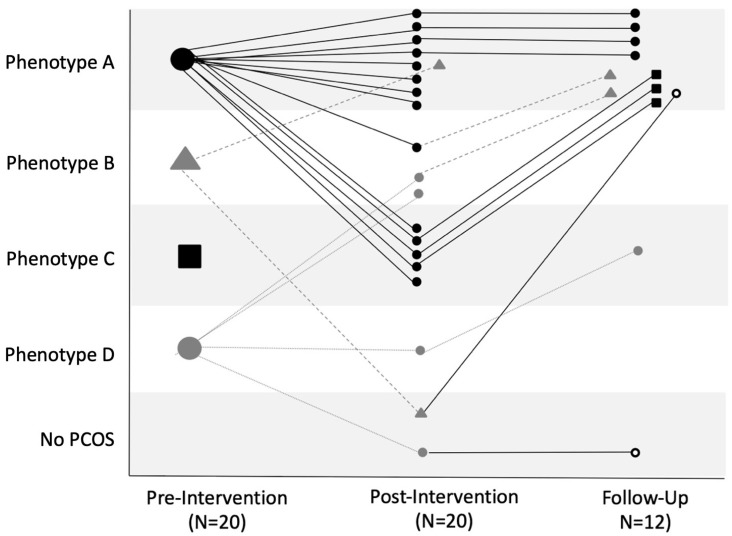
Individual trajectories in PCOS phenotype status at pre-intervention, post-intervention, and 6-month follow-up. Each line represents an individual participant’s phenotype status at pre-intervention (N = 20) to post-intervention (N = 20) and at the follow-up assessment (N = 12). The phenotypes are designated as follows: Phenotype A (black circle), Phenotype B (gray triangle), Phenotype C (black square), Phenotype D (gray circle), and No PCOS (open circle), with the phenotype shape shown as the previous assessment.

Figure 2 shows individual trajectories in PCOS phenotype across pre-intervention, post-intervention and 6-month follow-up stages. At pre-intervention, 14 participants met the criteria for Phenotype A, 2 were classified as Phenotype B, and 4 were considered Phenotype D. Post-intervention, the number of participants classified as Phenotype A was reduced to nine. Three participants now classified as Phenotype B, whereas only one participant had Phenotype D post-intervention. Phenotype C was now apparent in five participants, and two participants lost their PCOS status post-intervention. Of those that returned for the follow-up evaluation 6 months later, 10 were classified as Phenotype A, 1 as Phenotype C, and the remaining participant did not meet criteria for PCOS status. Four participants had remained in their Phenotype A designation since the post-intervention assessment, while others had transitioned to Phenotype A from Phenotype B (two participants), Phenotype C (three participants), and No PCOS (one participant) status. The participant with Phenotype C had transitioned from Phenotype D, while the remaining participant had persisted in no longer meeting the criteria for PCOS status.

Eight participants were designated as demonstrating a favorable change in PCOS phenotype from pre- to post-intervention stages, and 12 participants were designated as having experienced an unfavorable change. Table 3 contrasts baseline clinical, endocrine, and morphologic features across those with favorable versus unfavorable changes in PCOS phenotype. There were no differences in pre-intervention characteristics between those that had favorable versus unfavorable changes in PCOS phenotype. No clinical, endocrine, or morphologic feature at pre-intervention was predictive of a favorable change in PCOS phenotype following the hypocaloric dietary intervention (Table 4). Likewise, no degree of change in any clinical, endocrine, or morphologic feature at post-intervention was predictive of a favorable change in PCOS phenotype (Table 4).

## 4. Discussion

This study aimed to evaluate the impact of a short-term hypocaloric dietary intervention on the clinical presentation of PCOS. We had hypothesized that weight loss resulting from a 3-month hypocaloric dietary intervention would have sufficiently favorable impacts on menstrual cyclicity, hyperandrogenism, and/or polycystic ovarian morphology to elicit positive shifts in the phenotypic presentation of PCOS. Our approach was novel in including comprehensive and serial assessments of all three cardinal features of PCOS, in addition to considering the potential for lasting effects of the intervention 6 months later. We found that weight loss through a hypocaloric dietary intervention conferred positive changes in PCOS phenotype status in some but not all women with PCOS, with shifts in phenotype driven by improvements in the degree of menstrual cycle irregularity and follicle excess. Namely, 40% of the study participants demonstrated a favorable change in PCOS phenotype, with two (10%) losing their PCOS designation entirely at the post-intervention stage. There were no differences in baseline characteristics between those that had favorable versus unfavorable phenotypic shifts post-intervention. Likewise, changes in clinical features during the intervention did not associate with a change to a more favorable phenotype. At the 6-month follow-up, a majority of the participants had reverted to a more severe PCOS phenotype, consistent with the impact of the hypocaloric diet being somewhat transient. The 2023 International Evidence-based Guideline for the Assessment and Management of PCOS supports the effectiveness of lifestyle intervention for improvement of weight and metabolic health in women with PCOS [2]. Our findings align with this recommendation, as the participants in this study all experienced clinically meaningful changes in weight and body composition in response to a short-term hypocaloric diet. A range of diets have been evaluated in the context of PCOS, which include but are not limited to low-carbohydrate, low glycemic index/glycemic load, high protein, and Dietary Approaches to Stop Hypertension (DASH) diets [39]. The most recent evidence synthesis on the efficacy of specific diets in PCOS management did not identify one diet as more beneficial for metabolic outcomes compared to others [2]. Current practice guidelines are prudent in indicating that the effectiveness of lifestyle intervention on reproductive health outcomes is uncertain due to an inability to harmonize data [27]. To date, studies evaluating the impact of hypocaloric dietary interventions in women with PCOS have not consistently shown positive changes on markers of ovulatory dysfunction [40,41,42]. Data on frequency of ovulation or menstrual cyclicity are difficult to harmonize, limiting the ability to perform pooled analyses on the effectiveness of lifestyle intervention on ovulation rate, menstrual cyclicity, and likelihood of spontaneous pregnancy [2,43]. In this study, we leveraged self-reported data on menstrual cycle length in the previous year, alongside objective data on ovulatory frequency during a 4-month clinical trial, to show that weight loss secondary to a hypocaloric dietary intervention reduced menstrual cycle length. Reductions in average menstrual cycle length were sufficient to transition 30% of the participants into an ovulatory phenotype (type C), wherein none had met those criteria at the pre-intervention stage. As mentioned, a decrease in menstrual cycle length coincides with the findings of some but not all trials investigating the impact of hypocaloric dietary intervention on PCOS symptoms [44,45]. That such a notable number of women could expect a relatively rapid normalization of menstrual cycle status is compelling and has implications for improved fertility in the short-term, which is an important concern for women living with PCOS.

Ovarian dysmorphology also improved following hypocaloric dietary intervention. Namely, we noted a decrease in the total number of follicles present within each ovary, consistent with an improvement in follicle excess post-intervention. We have previously shown that follicle excess in PCOS is a consequence of excessive recruitment and not follicular persistence [46], yet the mechanisms underlying propensity for follicle excess in PCOS are ill-defined [47]. Increased local testosterone production secondary to inherent abnormalities in steroidogenic enzymes are posited to drive excessive activation of the primordial follicle pool in a manner that results in the stockpiling of primary follicles [48,49]. Few studies have assessed ovarian morphology following a lifestyle intervention in women with PCOS. We are aware of one study that showed lower follicle counts in women with PCOS randomized to a pulse-based diet [41], while another study showed decreased follicle counts on magnetic resonance imaging in those undergoing an exercise-based intervention [50]. Changes in ovarian morphology in this study were primarily restricted to follicle counts, as OV and AMH levels were unchanged following the hypocaloric dietary intervention. Ovarian size has been shown to decrease in women with PCOS after more extreme weight loss induced by bariatric surgery [51]. By contrast, changes in AMH with weight loss are controversial [52,53]. The lack of a change across all markers of polycystic ovarian morphology suggests that the changes in follicle counts post-intervention may have little clinical significance. Indeed, average FNPO post-intervention still met criteria for polycystic ovaries [2]. As such, the changes in follicle counts demonstrated in this study were only sufficient to drive phenotype shifts in a minority of the participants.

Consistent with our findings, we had anticipated that a short-term hypocaloric dietary intervention would not improve clinical markers of hyperandrogenism. Several studies evaluating hirsutism following a lifestyle change have noted little to no improvement in the degree of hirsutism post-intervention [54,55,56]. Lack of efficacy reflects the physiology of the cutaneous hair follicle, whose transition to terminal hair is governed by androgens and is largely irreversible [57]. That said, we had expected that weight loss would improve metabolic status, thereby increasing hepatic production of SHBG and decreasing free androgens. Indeed, short-term hypocaloric dietary intervention was effective at increasing SHBG, but this increase was not sufficient to offset a rise in serum testosterone post-intervention. The higher total testosterone levels post-intervention were unexpected. Most dietary intervention studies in PCOS show evidence of no change or a decrease in biochemical indicators of androgen excess, presumably owing to a decrease in the co-gonadotropin actions of insulin and the aforementioned increase in SHBG [58,59,60]. Insulin levels were unchanged by the hypocaloric dietary intervention. Rather, the increase in testosterone observed in this study was accompanied by an increase in LH, which is a primary driver of thecal cell androgen biosynthesis. Obesity is known to have a suppressive effect on gonadotropin production, and LH levels can rebound with calorie restriction and weight loss in women with PCOS [61,62,63]. Likewise, a change in diet composition could have contributed to altered testosterone production, as others have shown that dietary protein alone can impact androgen production [39]. Ultimately, hyperandrogenism was the cardinal feature most resistant to improvement by hypercaloric dietary intervention, and all but one participant with an androgenic PCOS phenotype transitioned to normoandrogenic status.

Most of women in this study presented with the most severe manifestation of PCOS, consistent with data on the prevalence of PCOS phenotypes [3,20]. Weight loss following a hypocaloric dietary intervention was not sufficient to alter phenotype in the majority of women with Phenotype A. However, four participants with Phenotype A did transition to more regular cycles with Phenotype C post-intervention. We are aware of one other study that examined phenotypic presentation of PCOS following a lifestyle intervention [29]. Similar to our study, this 12-month randomized controlled trial showed that women with a frank presentation of Phenotype A moved to the ovulatory Phenotype C during the lifestyle intervention [29]. However, in our study we did not demonstrate any participant with Phenotype A losing their PCOS status. That said, we could not identify any factor(s) at baseline that could predict a positive shift in PCOS presentation. Likewise, favorable changes in PCOS status did not align with the degree of change in any clinical, endocrine, or morphological feature evaluated. This inability to identify factors that predict response to treatment suggest that counselling related to the efficacy of hypocaloric diets on the clinical presentation of PCOS should be tempered for all women with PCOS regardless of phenotype at the onset. Last, the participants that returned for follow-up had regained weight compared to post-intervention, but weight still remained lower than at pre-intervention. The weight regain was likely owing to their cessation of consuming a hypocaloric commercial meal plan and elimination of frequent interactions with members of the research team, which can help with adherence to lifestyle change. Other studies that have included a follow-up assessment have reported increased energy intake and weight gain at follow-up but a continuation of menstrual cycle improvements [40,42]. The participants in our study largely reverted back to their original phenotype at the follow-up, with only one participant remaining without a PCOS designation. Although changes in phenotype appear to track with changes in weight, our study does not provide direct evidence of causality. Whether this type of remission is temporary or can be sustained is a novel topic for future research.

This study had several strengths. The participants were consistently evaluated using best practices for the assessment of ovulatory dysfunction, hyperandrogenism, and polycystic ovarian morphology. As such, our phenotyping for PCOS was robust at all timepoints of the study and did not lack assessments of ovarian morphology, which are most often missing in lifestyle intervention studies of PCOS. Our study was strengthened by the inclusion of a 6-month follow-up, which enabled an opportunity to assess the potential for lasting changes in PCOS phenotype following a feasible and effective short-term lifestyle intervention. An understanding of whether favorable changes in PCOS symptomology persist is needed to ensure counseling of the actual short- and long-term benefits of lifestyle intervention. This study was limited by its retrospective nature, single-arm study design, and the small sample size available for analysis. There was no a priori sample size calculation, as this represented an exploratory pilot analysis. With only 60% of the participants returning for follow-up, our assessments at this timepoint in particular can be considered exploratory. We acknowledge that our study population lacked racial and ethnic diversity and did not include those with significant metabolic disease progression (i.e., moderate to severe hypertension, dyslipidemia, or diabetes). As such, our findings are only generalizable to a subset of women with PCOS and cannot fully inform on the efficacy of hypocaloric intervention.

## 5. Conclusions

In conclusion, weight loss through a short-term hypocaloric dietary intervention can confer improvements in the phenotypic presentation of PCOS in some but not all women with PCOS. Improvements in PCOS phenotype are driven by improvements in menstrual cycle irregularity and follicle excess but not androgen excess. Factors promoting favorable shifts in the clinical presentation of PCOS are difficult to predict and are likely transient without maintenance of lifestyle modification. This pilot study provides a basis for future research to corroborate the potential of lifestyle intervention to prevent or reverse the progression of PCOS.

## Figures and Tables

**Table 1:** Anthropometric, body composition and metabolic status markers at pre- intervention, post intervention, and 6-month follow-up in women with PCOS undergoing a hypocaloric dietary intervention.

	Pre-Intervention (N = 20)	Post-Intervention (N = 20)	% Change Pre vs. Post	Follow-Up (N = 12)	% Change Post vs. Follow-Up
Weight (kg)	100.7 ± 21.2 ^a^	92.5 ± 18.7 ^b^	−8 ± 3	93.9 ± 23.1 ^c^	6 ± 3
BMI (kg/m^2^)	37.5 ± 6.0 ^a^	34.5 ± 5.4 ^b^	−8 ± 3	34.6 ± 5.9 ^c^	6 ± 3
Waist Circumference (cm)	108 ± 18 ^a^	96 ± 15 ^b^	−10 ± 4	97 ± 16 ^c^	7 ± 7
Hips Circumference (cm)	120 ± 13 ^a^	112 ± 12 ^b^	−7 ± 4	114 ± 13 ^a^	6 ± 4
Waist-to-Hips Ratio	0.88 ± 0.07 ^a^	0.86 ± 0.06 ^b^	−3 ± 5	0.84 ± 0.06 ^b^	0 ± 5
Total Percent Fat (%)	42 ± 5 ^a^	41 ± 4 ^b^	−3 ± 2	39 ± 7 ^ab^	2 ± 8
Lean-to-Fat Ratio	1.3 ± 0.3 ^a^	1.4 ± 0.3 ^b^	6 ± 5	1.5 ± 0.3 ^b^	−2 ± 5
Lean Mass (kg)	55.7 ± 8.7 ^a^	53.1 ± 8.1 ^b^	−6 ± 3	54.6 ± 9.9 ^a^	5 ± 3
Trunk Fat Mass (kg)	21.9 ± 7.5 ^a^	20.2 ± 6.4 ^b^	−10 ± 7	18.7 ± 8.2 ^ab^	7 ± 8
Systolic Pressure (mmHg)	123 ± 11 ^a^	117 ± 8 ^a^	−4 ± 9	122 ± 6 ^a^	8 ± 14
Diastolic Pressure (mmHg)	82 ± 8 ^a^	78 ± 6 ^a^	−4 ± 9	82 ± 5 ^a^	6 ± 10
Fasting Glucose (mg/dL)	96 ± 6 ^a^	94 ± 7 ^a^	−2 ± 7	95 ± 7 ^a^	2 ± 12
2 h Glucose (mg/dL)	113 ± 23 ^a^	104 ± 20 ^a^	−7 ± 21	112 ± 36 ^a^	2 ± 19
Fasting Insulin (ulU/mL)	19.6 ± 15.5 ^a^	14.6 ± 7.1 ^a^	−10 ± 36	15.6 ± 7.3 ^a^	29 ± 50
2 h Insulin (ulU/mL)	109.0 ± 110.6 ^a^	73.7 ± 40.3 ^a^	−12 ± 45	87.2 ± 59.9 ^a^	27 ± 66
HOMA-IR	4.8 ± 3.9 ^a^	3.4 ± 1.7 ^a^	−11 ± 37	3.7 ± 1.7 ^a^	30 ± 48

Linear mixed-models with Tukey HSD tests were used to compare endpoints across pre-intervention, post-intervention, and follow-up. Data are represented as a mean ± SD. Within rows, differences across timepoints are denoted by different letters (*p* < 0.05). Abbreviations: BMI, body mass index; HOMA-IR, homeostatic model assessment of insulin resistance.

**Table 2:** Diagnostic features of PCOS at pre-intervention, post-intervention, and 6-month follow-up in women with PCOS undergoing a hypocaloric dietary intervention.

	Pre-Intervention (N = 20)	Post-Intervention (N = 20)	% Change Pre vs. Post	Follow-Up (N = 12)	% Change Post vs. Follow-Up
Menstrual Cycle Length (d)	99 ± 77 ^a^	58 ± 32 ^b^	−20 ± 53	64 ± 26 ^ab^	38 ± 79
Regular (21–35 d)	0 (0%)	6 (30%)	-	1 (8.33%)	-
Mild Oligomenorrhea (36–59 d)	6 (30%)	7 (35%)	-	4 (33.33%	-
Oligomenorrhea (60–90 d)	8 (40%)	4 (20%)	-	5 (41.67%)	-
Amenorrhea (>90 d)	6 (30%)	3 (15%)	-	2 (16.57%)	-
LH (mlU/mL)	6.76 ± 3.29 ^a^	9.19 ± 4.12 ^b^	65 ± 114	6.77 ± 2.81 ^ab^	−9 ± 58
FSH (mlU/mL)	6.3 ± 1.9 ^a^	6.7 ± 1.9 ^a^	10 ± 27	6.3 ± 3.0 ^a^	−11 ± 36
LH: FSH ratio	1.1 ± 0.6 ^a^	1.4 ± 0.6 ^a^	44 ± 82	1.3 ± 0.9 ^a^	22 ± 110
Hirsutism Score	7 ± 4 ^a^	7 ± 4 ^a^	−3 ± 33	5 ± 3 ^a^	−6 ± 25
Total Testosterone (ng/dL)	49.8 ± 21.4 ^a^	83.3 ± 51.5 ^b^	105 ± 197	71.3 ± 18.5 ^b^	11 ± 37
SHBG (nmol/L)	24.2 ± 9.8 ^a^	28.5 ± 11.9 ^b^	23 ± 43	29.3 ± 12.9 ^ab^	−10 ± 14
Free Androgen Index	9 ± 6 ^a^	12 ± 11 ^a^	82 ± 199	11 ± 6 ^a^	27 ± 41
AMH (ng/mL)	8.7 ± 3.1 ^a^	9.2 ± 2.8 ^a^	10 ± 24	9.3 ± 3.0 ^a^	−0 ± 26
Mean FNPO	45 ± 33 ^a^	35 ± 20 ^b^	−10 ± 37	48 ± 31 ^ab^	32 ± 41
Mean OV (mL)	11.85 ± 4.36 ^a^	11.28 ± 3.83 ^a^	−3 ± 16	11.77 ± 7.10 ^a^	−5 ± 33

Linear mixed-models with Tukey HSD tests were used to compare endpoints across pre-intervention, post-intervention, and follow-up. Data are represented as a mean ± SD. Within rows, differences across timepoints are denoted by different letters (*p* < 0.05). Abbreviations: LH, Luteinizing Hormone; FSH, Follicle Stimulating Hormone; SHBG, Sex Hormone Binding Globulin; AMH, Anti-Mullerian Hormone; FNPO, Follicle Number Per Ovary; OV, Ovarian Volume.

**Table 3:** Baseline pre-intervention characteristics of participants with favorable versus unfavorable changes in PCOS phenotype following a hypocaloric dietary intervention.

Pre-Intervention Markers	Favorable (N = 8)	Unfavorable (N = 12)	*p*-Value
Age (y)	27 ± 4	28 ± 5	0.588
Baseline MCL	114 ± 111	89 ± 46	0.565
Weight (kg)	102.2 ± 25.3	99.6 ± 19.1	0.616
BMI (kg/m^2^)	37.7 ± 6.5	37.4 ± 5.9	0.925
Waist Circumference (cm)	107 ± 24	108 ± 14	0.728
Hips Circumference (cm)	119 ± 14	121 ± 12	0.761
Waist-to-hips Ratio	0.89 ± 0.10	0.89 ± 0.05	0.512
Systolic Pressure (mmHg)	123 ± 10	123 ± 11	0.898
Diastolic Pressure (mmHg)	81 ± 8	84 ± 8	0.434
Fasting Glucose (mg/dL)	94 ± 6	98 ± 6	0.232
2 h Glucose (mg/dL)	113 ± 28	114 ± 22	0.958
Fasting insulin (ulU/mL)	17.6 ± 13.4	21.0 ± 17.3	0.908
2 h insulin (ulU/mL)	84.7 ± 45.8	125.2 ± 138.2	0.362
HOMA IR	4.1 ± 3.1	5.1 ± 4.4	0.787
Lean: Fat Ratio	1.4 ± 0.4	1.3 ± 0.3	0.784
Total Percent fat (%)	42 ± 6	42 ± 4	0.932
Lean Mass (kg)	56.4 ± 10.4	55.3 ± 7.9	0.806
Trunk Fat Mass (kg)	21.8 ± 9.7	22.0 ± 6.2	0.967
LH (mlU/mL)	5.7 ± 3.3	7.5 ± 3.2	0.256
FSH (mlU/mL)	6.4 ± 2.5	6.3 ± 1.4	0.909
LH: FSH ratio	0.9 ± 0.5	1.3 ± 0.6	0.184
Hirsutism	6 ± 5	8 ± 4	0.533
Free Androgen Index	7 ± 4	9 ± 7	0.563
Total Testosterone (ng/dL)	49.9 ± 16.1	49.8 ± 25.1	0.440
SHBG (nmol/L)	26.0 ± 8.3	22.9 ± 10.8	0.483
AMH (ng/mL)	7.7 ± 3.9	9.5 ± 2.2	0.251
Mean OV (mL)	11 ± 4	13 ± 5	0.596
Mean FNPO (2–9 mm)	43 ± 29	46 ± 36	0.847

Data are represented as a mean ± SD. Abbreviations: LH, Luteinizing Hormone; FSH, Follicle Stimulating Hormone; SHBG, sex hormone binding globulin; AMH, Anti-Mullerian Hormone; FNPO, follicle number per ovary; OV, ovarian volume.

**Table 4:** Associations of baseline pre-intervention markers and percent change in markers post-intervention with favorable phenotype change in women with PCOS following a hypocaloric dietary intervention.

	Pre-Intervention			% Change		
Variable	β	Odds Ratio	95% CI	β	Odds Ratio	95% CI
Age (y)	−0.056	0.946	[0.776, 1.153]	-	-	-
Menstrual Cycle Length (d)	0.004	1.004	[0.992, 1.017]	−0.009	0.991	[0.972, 1.011]
Weight (kg)	0.006	1.006	[0.964, 1.050]	0.140	1.151	[0.833, 1.589]
BMI (kg/m^2^)	0.008	1.008	[0.865, 1.175]	0.147	1.159	[0.832, 1.612]
Waist Circumference (cm)	−0.004	0.996	[0.947, 1.048]	0.099	1.104	[0.869, 1.403]
Hips Circumference (cm)	−0.013	0.987	[0.917, 1.063]	0.204	1.227	[0.918, 1.639]
Waist-to-hips Ratio	−0.493	0.611	[2.07 × 10^−6^, 180,252.8]	−0.051	0.950	[0.775, 1.164]
Systolic Pressure (mmHg)	0.006	1.006	[0.922, 1.097]	−0.040	0.961	[0.863, 1.070]
Diastolic Pressure (mmHg)	−0.050	0.951	[0.845, 1.070]	0.022	1.022	[0.923, 1.133]
Fasting Glucose (mg/dL)	−0.100	0.905	[0.767, 1.067]	0.023	1.023	[0.893, 1.172]
2 h Glucose (mg/dL)	−0.001	0.998	[0.961, 1.039]	0.055	1.056	[0.995, 1.121]
Fasting Insulin (ulU/mL)	−0.016	0.985	[0.925, 1.048]	0.026	1.026	[0.996, 1.058]
2 h Insulin (ulU/mL)	−0.004	0.995	[0.984, 1.007]	0.025	1.026	[0.992, 1.060]
HOMA IR	−0.076	0.927	[0.715, 1.201]	0.023	1.024	[0.995, 1.053]
Lean: Fat Ratio	0.327	1.387	[0.070, 27.452]	0.197	1.218	[0.945, 1.570]
Total Percent Fat (%)	−0.009	0.991	[0.821, 1.195]	−0.367	0.693	[0.429, 1.120]
Lean Mass (kg)	0.000	1.000	[1.000, 1.000]	0.392	1.480	[0.959, 2.282]
Fat Mass (kg)	−0.000	1.000	[1.000, 1.000]	−0.030	0.971	[0.847, 1.112]
LH	−0.186	0.830	[0.607, 1.134]	0.010	1.010	[0.996, 1.025]
FSH	0.035	1.036	[0.631, 1.701]	0.028	1.028	[0.983, 1.075]
LH: FSH ratio	−1.230	0.292	[0.042, 2.032]	0.013	1.013	[0.992, 1.034]
Hirsutism	−0.075	0.928	[0.745, 1.156]	−0.011	0.989	[0.960, 1.019]
Free Androgen Index	−0.066	0.936	[0.786, 1.114]	−0.004	0.996	[0.986, 1.005]
Total Testosterone (ng/dL)	0.000	1.000	[0.959, 1.004]	−0.010	0.991	[0.975, 1.007]
SHBG (nmol/L)	0.034	1.035	[0.940, 1.389]	−0.039	0.962	[0.907, 1.019]
AMH_PICO	−0.000	1.000	[1.000, 1.000]	0.023	1.023	[0.980, 1.068]
Mean FNPS	0.066	1.069	[0.887, 1.287]	−0.007	0.993	[0.979, 1.008]
Mean OV (mL)	−0.055	0.947	[0.776, 1.155]	0.008	1.008	[0.951, 1.069]
Mean FNPO (2–9 mm)	−0.004	0.996	[0.968, 1.026]	−0.008	0.992	[0.966, 1.019]

β estimates, unit odds ratio and 95% confidence interval of baseline and percent change of each variable. Abbreviations: LH, Luteinizing Hormone; FSH, Follicle Stimulating Hormone; SHBG, sex hormone binding globulin; AMH, Anti-Mullerian Hormone; FNPO, follicle number per ovary; OV, ovarian volume.

## Data Availability

The data and materials used for the current study are available from the corresponding author upon reasonable request. The data are not publicly available due to privacy reasons.
